# Methylmercury Effects and Exposures: Who Is at Risk?

**DOI:** 10.1289/ehp.1205357

**Published:** 2012-06-01

**Authors:** Celia Chen

**Affiliations:** Department of Biological Sciences, Dartmouth College, Hanover, New Hampshire, E-mail: celia.chen@dartmouth.edu

Two articles in this issue of *EHP* represent recent syntheses of research on the effects of mercury exposure from fish consumption: Karagas et al. (2012) reviewed the emerging research on health effects of low-level exposures to methylmercury (MeHg), and Oken et al. (2012) summarized the complexities of providing clear and uniform fish consumption advice to reduce MeHg exposure while balancing nutrient intake, ecologic concerns, and economic issues. These two papers emerged from workshops convened in September 2010 and July 2011 by the Coastal Marine Mercury Ecosystem Research Collaborative (C-MERC) and sponsored by the Dartmouth Superfund Research Program and its partners. C-MERC brought together a group of 50 scientists and stakeholders to work jointly to gather and analyze existing data and to publish synthesis papers on the fate of mercury from its environmental sources to seafood consumers—issues of critical importance for informing public policy.

Mercury, particularly its organic form (MeHg), is a global contaminant and toxicant of major concern for humans and wildlife (Driscoll et al. 2007; Fitzgerald et al. 2007; Grandjean et al. 2005; Mahaffey et al. 2009). Mercury is third (after arsenic and lead) on the 2011 Agency for Toxic Substances and Disease Registry (ATSDR) priority  list of 275 hazardous substances (ATSDR 2011), which includes substances that present the most significant potential threats to human health in the United States. MeHg has long been known as a potent neurotoxicant, particularly due to incidents of acute and high-level exposures (e.g., Minimata, Japan; Iraq), but neurological effects have been documented in island populations that consume large quantities of marine mammals or seafood (Axelrad et al. 2007; Cohen et al. 2005; Rice 2004). Moreover, recent epidemiologic studies have revealed evidence of a range of health effects in adults and children at MeHg exposure levels lower than previously observed (Lynch et al. 2010; Mergler et al. 2007; Oken et al. 2008). In this issue of *EHP*, Karagas et al. (2012) provide a comprehensive review of the current scientific evidence for effects of low-level exposures to MeHg on birth outcomes, neurocognitive outcomes, the cardiovascular system, and immune function. The authors recommend that future  studies investigate sex-specific effects and genetic susceptibility, and that they include more precise exposure indicators, outcome measures with mechanistic bases, and consideration of nonlinear dose–response relationships. Their review helps to set the stage for future research on the human effects of low-level MeHg exposure.

Fish are the most important agents of MeHg exposure for humans, and consumption of contaminated fish is a serious public health concern (Mahaffey et al. 2009; Oken et al. 2005; Sunderland 2007). Currently, all 50 U.S. states have fish advisories for inland and coastal waters, and states on the Atlantic coast, as well as Alaska and Hawaii, have statewide coastal advisories [U.S. Environmental Protection Agency (EPA) 2010]. Consumption of marine fish and shellfish is the primary means of human exposure to MeHg; approximately 92% of the global fish and shellfish harvest for human consumption is marine [United Nations Development Programme, United Nations Environment Programme (UNEP), World Bank, and World Resources Institute 2003], with the majority coming from coastal fisheries (Food and Agriculture Organization of the United Nations 2010). Most people trying to reduce MeHg exposure risk do so through their choices in buying and eating seafood. Oken et al. (2012) discuss the wide range of trade-offs facing fish consumers and the difficulties in evaluating current fish consumption advice. Consumers need to consider not only the contaminant concentrations in fish but also their nutritional value, the sustainability of the fishery, and the cost of different fish choices. Moreover, there is little guidance for specific subpopulations with different exposure risks due to factors such as age or baseline intake of fish. The authors recommend that fish consumption advice address these multiple perspectives and provide a clear and simple message. Ultimately, fish consumption advice should protect public health on a global scale and promote sustainability of the world’s fisheries as a critical source of human nutrition.

Currently, important national and international policy decisions are being made concerning the environmental impacts of mercury. The widespread threat to human health posed by MeHg has prompted the United States to pass a mercury rule for controlling atmospheric emissions (U.S. EPA 2011) and the UNEP to forge a broad consensus among 140 participating countries to control mercury contamination through a global, legally binding mercury agreement (UNEP 2009). Moreover, U.S. Senate and House bills have been introduced in order to establish a national mercury monitoring network to track long-term trends in mercury levels in the atmosphere and in terrestrial, freshwater, and coastal ecosystems (Comprehensive National Mercury Monitoring Act 2011a, 2011b). To provide a synthesis of current marine mercury science to inform policy making, C-MERC stakeholders identified the major environmental and health policy questions associated with MeHg, and C-MERC scientists reviewed and summarized the current scientific knowledge available for addressing those questions. In addition to the two articles in this issue of *EHP*, additional papers will be published in an upcoming special issue of *Environmental Research* on the fate of MeHg in estuaries, coastal oceans, and the open ocean. The goal of all of these reports is to provide scientists and policy makers with an understanding of the links between environmental processes that affect MeHg levels in aquatic ecosystems and human MeHg exposure and health risks. These links are critical to predicting how local and global changes in environmental mercury levels will ultimately influence MeHg contamination of seafood and the resulting human exposure risk.
